# Cell-Free DNA Tumor Fraction in the Aqueous Humor Is Associated With Therapeutic Response in Retinoblastoma Patients

**DOI:** 10.1167/tvst.9.10.30

**Published:** 2020-09-30

**Authors:** Ashley Polski, Liya Xu, Rishvanth K. Prabakar, Jonathan W. Kim, Rachana Shah, Rima Jubran, Peter Kuhn, David Cobrinik, James Hicks, Jesse L. Berry

**Affiliations:** 1 The Vision Center at Children's Hospital Los Angeles, Los Angeles, CA, USA; 2 USC Roski Eye Institute, Keck School of Medicine of the University of Southern California, Los Angeles, CA, USA; 3 Department of Biological Sciences, Dornsife College of Letters, Arts, and Sciences, University of Southern California, Los Angeles, CA, USA; 4 Department of Molecular and Computational Biology, University of Southern California, Los Angeles, CA, USA; 5 Cancer and Blood Disease Institute at Children's Hospital Los Angeles, Los Angeles, CA, USA; 6 Norris Comprehensive Cancer Center, Keck School of Medicine, University of Southern California, Los Angeles, CA, USA; 7 Department of Aerospace and Mechanical Engineering, Viterbi School of Engineering, University of Southern California, Los Angeles, CA, USA; 8 Department of Biomedical Engineering, Viterbi School of Engineering, University of Southern California, Los Angeles, CA, USA; 9 Department of Biochemistry and Molecular Medicine, Keck School of Medicine, University of Southern California, Los Angeles, CA, USA; 10 The Saban Research Institute, Children's Hospital Los Angeles, Los Angeles, CA, USA

**Keywords:** retinoblastoma, aqueous humor, liquid biopsy, tumor fraction, somatic copy number alteration

## Abstract

**Purpose:**

The aqueous humor (AH) liquid biopsy enables in vivo evaluation of tumor-derived cell-free DNA (cfDNA) from retinoblastoma (RB) eyes. Herein, we test our hypothesis that longitudinal dynamics of AH cfDNA—including tumor fraction (TFx) and somatic copy number alteration (SCNA) amplitude—correspond to therapeutic response.

**Methods:**

Eyes with ≥3 AH extractions during intravitreal chemotherapy (IVM) or at secondary enucleation between 2015 to 2019 were included. AH cfDNA was sequenced to assess RB SCNA amplitude; ichorCNA software was used to estimate TFx. Eyes without SCNAs or with TFx < 0.10 across all samples were excluded. Therapeutic responses for each eye were determined from clinical records. Statistical analyses included Mann-Whitney U and Pearson correlation tests.

**Results:**

Twenty eyes of 20 patients underwent ≥3 AH extractions; 6 eyes lacked SCNAs or had TFx < 0.10 throughout sampling and were excluded. Clinical progression was associated with significantly higher SCNA amplitudes and TFx values than regression (*P* ≤ 0.04). Relative increases in TFx (ΔTFx 1.86 ± 2.22) were associated with disease progression, whereas relative decreases in TFx (ΔTFx 0.53 ± 0.36) were associated with disease regression (*P* < 0.00001). A ≥15% increase in TFx relative to baseline during treatment was associated with an over 90-fold increased likelihood of clinical progression (odds ratio = 90.67, 95% confidence interval = 8.30–990.16, *P* = 0.0002). TFx and SCNA amplitude were significantly positively correlated throughout sampling (*P* ≤ 0.002).

**Conclusions:**

Longitudinal changes in AH-derived cfDNA TFx and SCNA amplitude are concordant with clinical responses of intraocular RB during active therapy.

**Translational Relevance:**

Longitudinal evaluation of AH cfDNA may provide an objective, quantitative way to monitor therapeutic response and disease burden in RB patients.

## Introduction

Retinoblastoma (RB) is a childhood cancer that forms in the developing retina. Although tumor biopsy is the diagnostic norm for many malignancies, direct biopsy is contraindicated in the setting of RB because of the risk of provoking extraocular tumor seeding and orbital relapse.^1^^–^^7^ Currently, tumor tissue is only available for molecular and genetic analyses from enucleated eyes.^8^ This inherently limits our understanding of in vivo RB dynamics and the relationship between molecular markers of RB and therapeutic response. Given the lack of in vivo molecular information, our ability to monitor RB activity during active therapy relies almost exclusively on imaging and clinical observations by the treating ocular oncologist. Because of this, young patients with RB often require monthly examinations under anesthesia as surveillance for persistent or recurrent disease. In current practice, there is no gold-standard objective, quantitative way to monitor intraocular dynamics or tumor load of RB throughout longitudinal clinical care.

Our recent development of an aqueous humor (AH) liquid biopsy was the first research-based modality to address the lack of direct tumor biopsy by facilitating in vivo sampling of tumor-derived cell-free DNA (cfDNA).^8^^–^^10^ Unlike traditional biopsies, a liquid biopsy allows for minimally invasive investigations of tumor-derived biomarkers in real time.^11^ Through our novel liquid biopsy platform, we demonstrated that AH is a high-yield source of RB cfDNA, that the cfDNA is concordant with corresponding genomic profiles of the tumor,^8^^,^^9^ and that tumor-derived somatic copy number alterations (SCNAs) are predictive of ocular survival.^8^ We also identified two cases in which changes in the amplitude of highly-recurrent RB SCNAs correlated with clinical tumor response.^8^ However, on a larger scale it remains unknown whether longitudinal AH sampling could be reliably used to monitor therapeutic response of RB over time.

The concept of using a liquid biopsy to monitor malignant disease is not new in the field of oncology. Numerous studies of nonocular cancers have associated the dynamics of circulating cfDNA in plasma with therapeutic response.^12^^–^^16^ Tumor fraction (TFx)—the proportion of sampled cfDNA that is tumor-derived—has demonstrated particular promise as a diagnostic and prognostic biomarker. In cancers of the prostate and lung, for example, cfDNA TFx in the blood was found to correspond with clinical therapeutic response^12^^,^^13^; in some cases, TFx was better able to indicate an early recurrence of disease than clinical observation or imaging alone.^13^ If a similar longitudinal relationship between AH cfDNA dynamics and RB tumor activity exists, then the AH liquid biopsy could potentially expand our understanding of tumor evolution, help clinicians to identify intraocular recurrences before they are clinically apparent (in conjunction with imaging modalities such as optical coherence tomography and B-scan ultrasonography), and allow us to generate precision-based approaches to RB management.

In this study, we evaluate our hypothesis that the longitudinal dynamics of AH cfDNA (specifically SCNA amplitude and TFx) are concordant with clinical therapeutic responses in eyes with RB. Through retrospective analysis of longitudinal AH samples from RB eyes, we demonstrate that changes in SCNA amplitude and TFx correspond to clinical progression and regression of intraocular disease, and that TFx dynamics within the AH are predictive of clinical response in RB eyes.

## Methods

### Study Approval

This retrospective observational study was conducted with Children's Hospital Los Angeles (CHLA) Institutional Review Board approval and adhered to the tenets of the Declaration of Helsinki. Before inclusion, written informed consent was obtained from the parents of all participants.

### Clinical Participants and Therapeutic Response

All patients who required intravitreal melphalan (IVM) for vitreous tumor seeding and had three or more sequential AH samples extracted during active IVM therapy or at secondary enucleation between 2015 to 2019 were included. Patients treated with IVM who did not have AH extracted—or who had fewer than three AH extractions—were excluded. Additional RB treatment during the study (including systemic chemotherapy, intraarterial chemotherapy, or secondary enucleation) was carried out in a nonrandomized manner per CHLA protocol.^17^^,^^18^

Retrospective chart review of each participant was performed to determine the therapeutic response of eyes with RB at the time of each AH extraction. All clinical descriptions of disease were based on operative reports from fundoscopic examinations under anesthesia, which were performed and recorded by one of two ocular oncologists. The therapeutic responses of eyes (clinical progression or regression) were determined both (1) relative to baseline (i.e., the time of first AH extraction) and (2) relative to the previous sampling. Specifically, eyes with a relative increase in vitreous seeding, tumor growth, appearance of new preretinal tumors secondary to active seeding, or recurrence were considered progressed; whereas eyes with a reduction or stability in active seeding or reduced tumor size were considered regressed. The retrospective determination of disease progression or regression at each sampling timepoint was confirmed by an ocular oncologist. Retrospective chart review was blinded to molecular testing results, because the genetic and genomic findings from AH samples were kept separate from clinical findings until final statistical analysis.

### Clinical Specimens

As described previously, 0.1-mL samples of AH were extracted via clear cornea paracentesis.^8^^–^^10^ AH extraction occurred either (1) during routine intravitreal therapy for seeding as part of the standard IVM injection protocol^19^ or (2) immediately after secondary enucleation of the eye; per IRB guidelines, AH was not taken at any time outside of these contexts.^8^^,^^9^ According to protocol, the scleral injection site for IVM was treated with cryotherapy; the corneal paracentesis site was not. Specimens were stored at −80°C immediately after extraction and underwent cfDNA isolation using the QIAamp Circulating Nucleic Acid Kit (Qiagen, Hilden, Germany).^8^^–^^10^

### CfDNA Library Construction and Sequencing

Library construction and sequencing of AH cfDNA for SCNA analyses were previously described in detail.^8^^–^^10^^,^^20^^,^^21^ Briefly, isolated cfDNA was constructed into whole genome libraries using the QIAseq Ultralow Input Library Kit (Qiagen). Within one month of extraction, libraries were then sequenced on an Illumina HiSeq (Illumina, San Diego, CA, USA) platform using the single-end 50 base pair (bp) sequencing mode.

### Determination of SCNA Amplitude

SCNA analyses were performed according to our previously described protocol.^8^^–^^10^^,^^20^^,^^21^ Copy number values were recorded as ratios to the median (relative to a baseline human genome), with values ≤0.87 representing copy number losses and values ≥1.15 representing copy number gains.^8^^–^^10^ Genomic profiles were evaluated to identify the presence of highly-recurrent RB SCNAs (including gains of 1q, 2p, and 6p, and losses of 13q and 16q)^22^ in each eye. For visualization purposes, whether there was a gain or a loss, SCNA *absolute* amplitudes—determined by |1 − ratio to the median|—were followed up longitudinally for each eye. It should be noted that changes in SCNA amplitude at a specific locus can be influenced not only by the actual copy number at that locus, but also by the overall proportion of cfDNA that originates from tumor cells, as discussed previously.^8^

### Determination of cfDNA TFx

The fraction of tumor DNA (TFx) for each sequenced AH cfDNA sample was estimated using ichorCNA software (available at https://github.com/broadinstitute/ichorCNA), a standard tool for determining cfDNA TFx in blood-based liquid biopsies.^23^ The algorithm used by ichorCNA to determine TFx of cfDNA in the serum has been described in detail.^12^^,^^23^ In short, ichorCNA uses a hidden Markov model to predict large-scale SCNAs within sequenced cfDNA. TFx estimations based on the presence of SCNAs are derived while accounting for differences in ploidy and subclonality at each locus, and an optimal TFx solution (as well as numerous alternative solutions) is provided by ichorCNA.^23^ Per ichorCNA recommendations, genomic profiles and corresponding TFx solutions were visually inspected to confirm the TFx estimate for each sample (htttps://github.com/broadinstitute/ichorCNA/wiki/Interpreting-ichorCNA-results). If the selected solution appeared suboptimal based on ichorCNA guidelines (i.e., a large proportion of SCNAs were being called subclonal, the majority of datapoints were falsely amplified, or two distinct copy number levels were being called neutral),^23^ then a more accurate alternative solution was manually selected from the ichorCNA output (available upon request).

### Statistics

The following were excluded from final statistical analysis: (1) eyes without SCNAs in any sample (ichorCNA depends on the detection of SCNAs to derive accurate TFx estimations^23^) or (2) eyes with a sustained TFx < 0.10 (the recommended threshold for standard depths of whole exome sequencing of cfDNA^23^) in all samples. The Mann-Whitney U test was used to assess for associations between (1) SCNA amplitude and therapeutic response and between (2) TFx and therapeutic response. Pearson correlation was used to determine the association between TFx and absolute SCNA amplitudes. *P* values <0.05 were considered significant.

## Results

### Clinical Participants and Therapeutic Response

Twenty eyes of 20 RB patients had ≥3 AH extractions during the study period, for a total of 78 AH samples. No AH samples were excluded from the study prior to sequencing and genomic analysis (sequencing quality metrics provided in Supplementary Table S1). Six eyes (30%) had no SCNAs or had TFx < 0.10 throughout all of their samples and were excluded from final statistical analysis (patient demographics and clinical characteristics of these six eyes are shown in Supplementary Table S2). For the remaining 14 eyes, demographics, clinical information, and AH findings are summarized in Table 1; case numbers are as per our prior studies for consistency.^8^ Of these 14 eyes, the median number of AH samples per eye was 4 (range 3–7 samples per eye) and included a total of 60 samples. The median interval between AH extractions was 3.5 weeks (range 1–72 weeks) (see Table 1). One eye (Case 15) had an interval of 72 weeks between its third and fourth samples (with a massive tumor recurrence noted at the fourth and final sample), although all other samples in the study were separated by 18 weeks or less. Fifty-seven (95%) of the 60 samples were extracted during IVM injection for tumor seeding, and three samples (5%) were extracted immediately after secondary enucleation in eyes that had previously undergone IVM with AH extraction. Of the 14 eyes included in analysis, clinical progression relative to baseline was identified at 11 different sampling timepoints in a total of 6 eyes. In all sampling timepoints except for one (the final sample of Case 6; see Table 1), therapeutic response (progression vs. regression) *relative to the preceding sample* matched therapeutic response relative to baseline. No participant had complications secondary to AH extraction with an average follow-up of 16.6 months from last injection (range 1–45), and none have developed extraocular or metastatic disease at any time in their follow-up.

**Table 1.** Clinical Demographics, Genomic Profiling of AH Samples, and Therapeutic Response for Each Study Participant

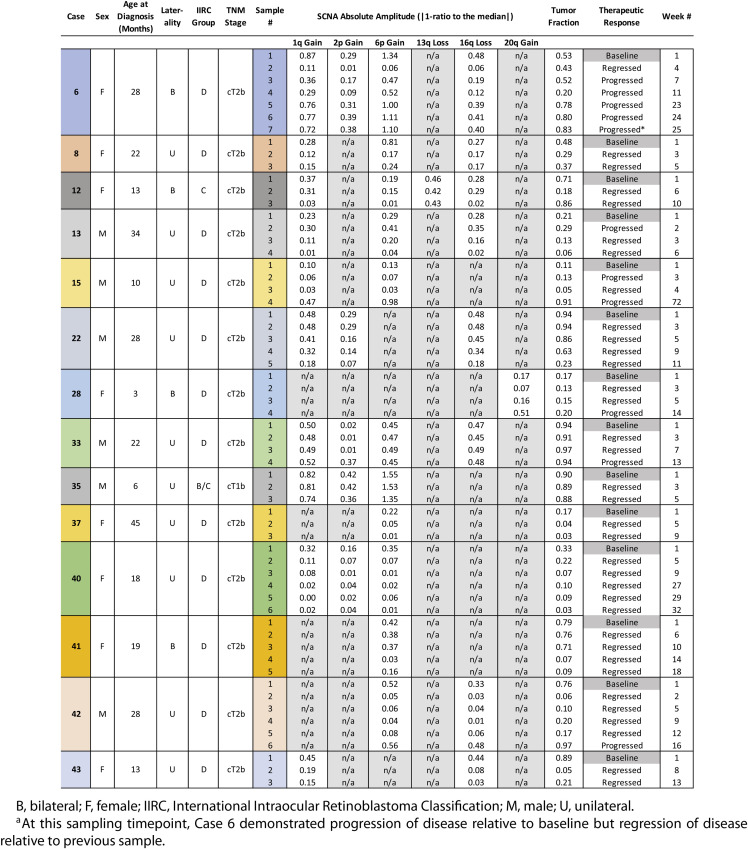	

B, bilateral; F, female; IIRC, International Intraocular Retinoblastoma Classification; M, male; U, unilateral.

a At this sampling timepoint, Case 6 demonstrated progression of disease relative to baseline but regression of disease relative to previous sample.

### SCNA Amplitude Corresponds to Clinical Therapeutic Response

Thirteen of the 14 eyes (92.9%) had at least one of the highly-recurrent RB SCNAs during sampling; these include gain of 1q, 2p, and 6p, and loss of 13q and 16q. One eye (Case 28) had a 20q gain but no highly recurrent RB SCNAs, so 20q amplitude was followed over time instead. As per previous reports,^8^^,^^9^^,^^22^ a gain of 6p was the most prevalent alteration (11/14 eyes, 78.8%), followed by 1q gain (10/14 eyes, 71.4%), 16q loss (8/14 eyes, 57.1%), 2p gain (5/14 eyes, 35.7%), 13q loss (1/14 eyes, 7.1%), and 20q gain (1/14 eyes, 7.1%). For the SCNAs that were present in more than one eye (including gain of 6p, 1q, and 2p, and loss of 16q), clinical progression (relative to baseline) at any sampling timepoint was associated with significantly higher SCNA amplitudes than clinical regression (*P* ≤ 0.04; see Table 2).

**Table 2.** Amplitudes of RB SCNAs Are Significantly Higher In the Setting of Clinical Progression (Relative to Baseline) Than Regression (*P* < 0.05)

	SCNA Amplitude (Mean ± SD)		
SCNA	Clinical Progression	Clinical Regression	*P* Value^a^
6p gain (11 eyes)	0.666 ± 0.353	0.228 ± 0.379	0.0008
1q gain (10 eyes)	0.472 ± 0.261	0.244 ± 0.233	0.043
16q loss (8 eyes)	0.351 ± 0.132	0.186 ± 0.175	0.032
2p gain (5 eyes)	0.283 ± 0.124	0.119 ± 0.139	0.019

SCNA amplitude is represented by the absolute amplitude—determined by |1 − ratio to the median|—for both copy number gains and copy number losses. SD, standard deviation.

a Mann-Whitney U test.

### TFx Corresponds to Clinical Therapeutic Response

The software ichorCNA was used to estimate AH cfDNA TFx based on the presence of SCNAs within sampled cfDNA.^23^ As above, 14/20 eyes (70%) had at least one AH sample with TFx ≥ 0.10 throughout the study period and were included in further analysis. For 19 of the 60 total samples (31.7%), the ichorCNA-selected TFx estimate was deemed suboptimal on visual inspection, and a more accurate estimate was manually chosen from ichorCNA output in accordance with ichorCNA recommendations.^23^

Of the 14 eyes included in analysis, median TFx for all AH samples was 0.29 (range 0.03–0.97). Clinical progression (relative to baseline) at any sampling timepoint was associated with a significantly higher TFx (mean 0.60 ± 0.34) than clinical regression (mean 0.34 ± 0.34; *P* = 0.022). Increases in TFx relative to baseline (ΔTFx 1.86 ± 2.22) were associated with disease progression, whereas decreases in TFx relative to baseline (ΔTFx 0.53 ± 0.36) were associated with disease regression (*P* < 0.00001). Similarly, when therapeutic response was defined *relative to the previous sample*, disease progression was associated with increases in TFx between samples (ΔTFx 3.46 ± 5.19), whereas disease regression was associated with general stability of TFx between samples (ΔTFx 0.99 ± 0.96; *P* = 0.006). A ≥15% TFx increase *between samples* was associated with an over eightfold increased likelihood of clinical progression (odds ratio [OR] = 8.17, 95% confidence interval [CI] = 1.71–39.03, *P* = 0.008), whereas an increase of 15% or more in TFx *relative to baseline* was associated with a more than 90-fold increased likelihood of clinical progression (OR = 90.67, 95% CI = 8.30–990.16, *P* = 0.0002).

### Dramatic Increases in SCNA Amplitude and TFx were Associated with a Clinical Recurrence

**Figure 1.**
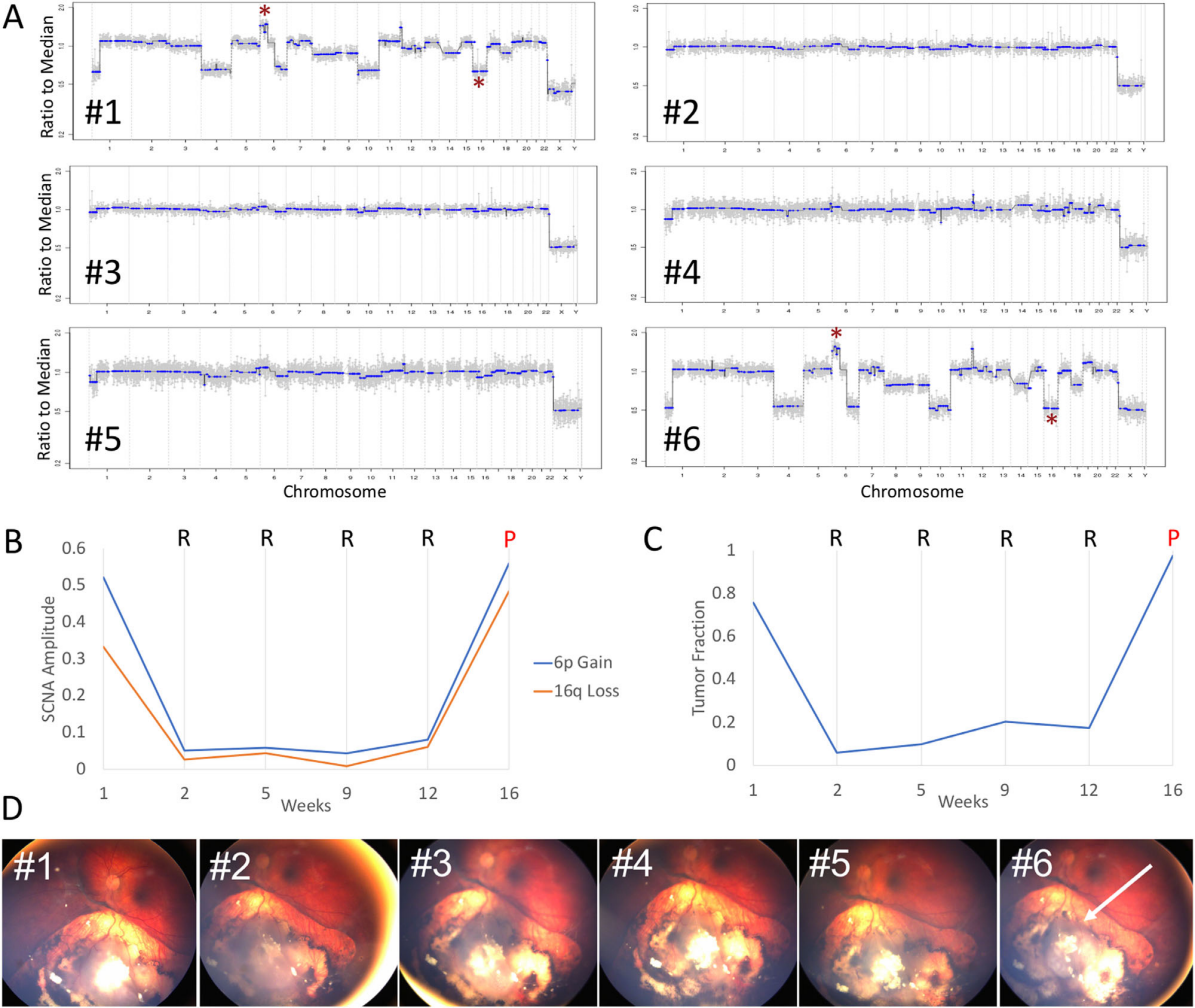
SCNA amplitude and TFx values correspond to clinical therapeutic response. (**A**) Genomic profiles of six sequential AH samples extracted from a single eye (case 42) during IVM therapy for tumor seeding (* = 6p gain or 16q loss). (**B**) Changes in SCNA amplitude correspond to clinical regression (*R*) and progression (*P*) at each sampling timepoint. (**C**) Changes in TFx correspond to clinical regression (*R*) and progression (*P*) at each sampling timepoint. (**D**) Fundus photographs of the affected eye over the course of 6 injections of IVM. Reduction of vitreous seeding and tumor size occurred between injections 2 through 5, whereas a large recurrence of the retinal tumor (*arrow*) was noted on the day of injection 6.

The relationship between AH cfDNA properties and clinical therapeutic response was particularly apparent in case 42, a 28-month-old male who presented with unilateral group D^24^/stage cT2b^25^ RB (Fig. 1). The affected eye underwent a total of six AH extractions in conjunction with six injections of IVM for tumor seeding (see Table 1). Within one week of initiating IVM (between injections 1 and 2), the absolute amplitudes of 6p gain and 16q loss decreased from 0.52 to 0.05 and 0.33 to 0.03, respectively (Figs. 1A, 1B); similarly, the TFx decreased from 0.76 to 0.06 (Fig. 1C). These changes were associated with concurrent regression of vitreous seeding and a shrinkage of the main retinal tumor on examination (Fig. 1D). Between samples 2 to 5 (over 10 weeks), intraocular disease continued to demonstrate apparent clinical regression (relative to both baseline *and* preceding samples) despite slight increases in SCNA amplitude (0.05 to 0.08 for 6p, 0.03 to 0.06 for 16q) and TFx (0.06 to 0.17) during this time. Specifically, the TFx at the time of sample 4 had doubled relative to the previous sample (ΔTFx 2.02), suggesting a more than eightfold likelihood of disease progression (as described above), although clinically there was no obvious disease activity at that time. Between the fifth and sixth injections, substantial increases in both SCNA amplitude (0.08 to 0.56 for 6p, 0.06 to 0.48 for 16q) and TFx (0.17 to 0.97) (Figs. 1B, 1C) coincided with a significant clinically apparent recurrence of the retinal tumor (Fig. 1D). Although an increase in TFx was noted at sample 4, no clinically evident tumor was noted on examination until the time of sample 6, highlighting the potential utility of assaying TFx to demonstrate preclinical tumor recurrences.

### TFx and SCNA Amplitude are Correlated

**Figure 2.**
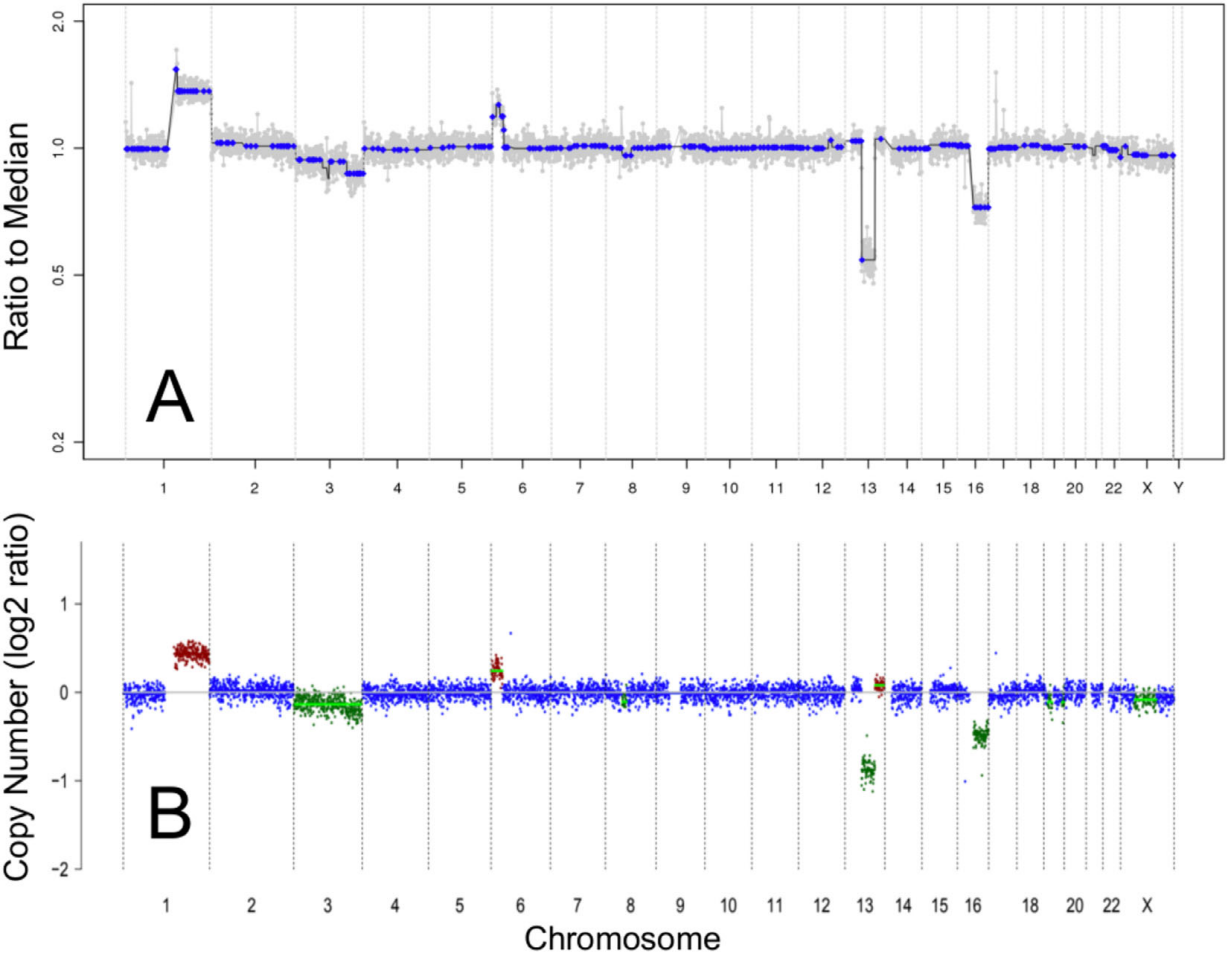
Genomic profiles showing SCNAs from cell-free DNA in the aqueous humor of an eye with retinoblastoma (case 12). The genomic profiles generated by our established SCNA analysis protocol (**A**) were concordant with the genomic profiles generated by the ichorCNA pipeline (**B**). In ichorCNA profiles, the color of each data point corresponds to estimated integer copy number (*green* = 1 copy, *blue* = 2 copies, *brown* = 3 copies, *red* = 4+ copies).

**Figure 3.**
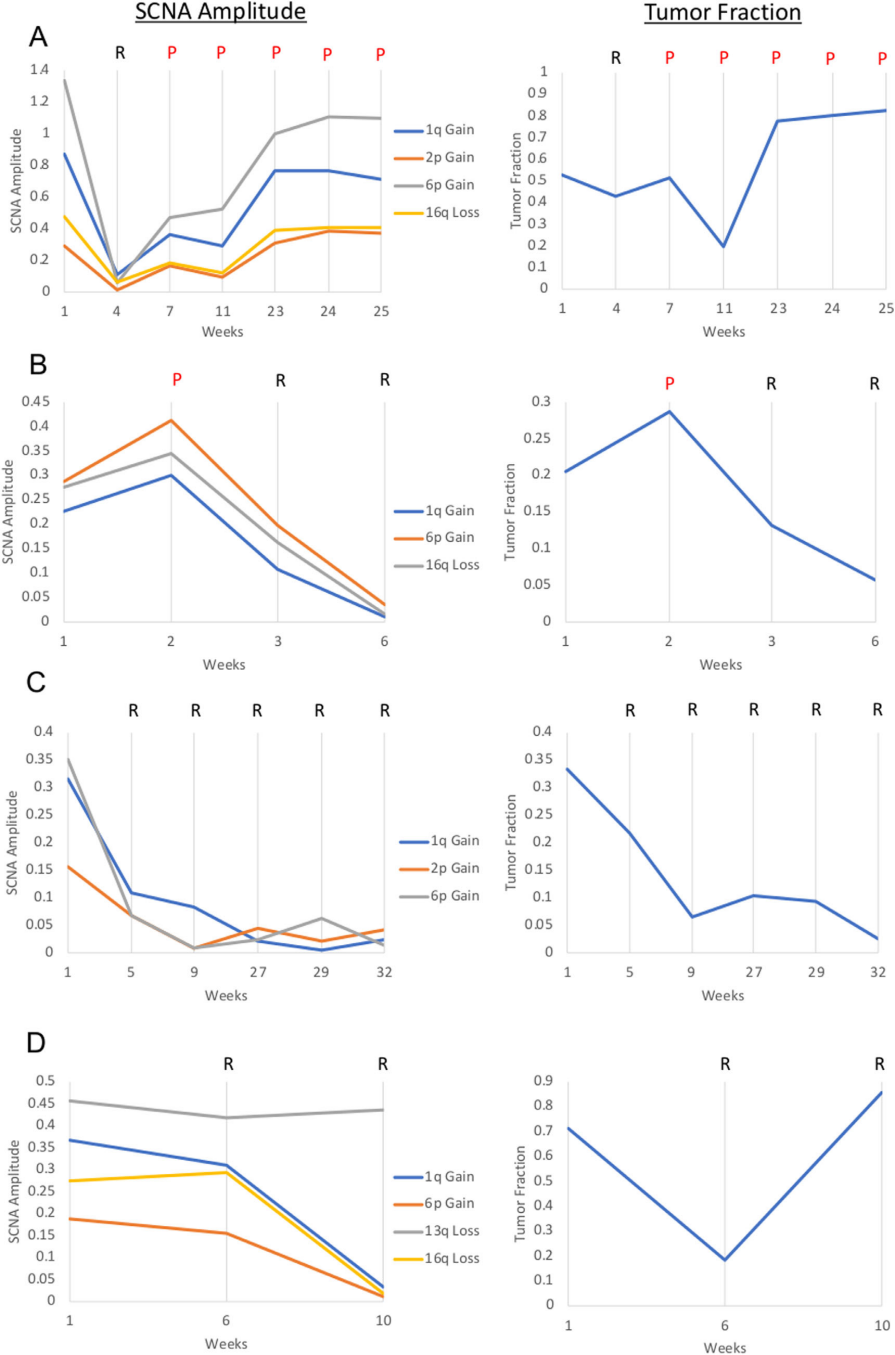
SCNA absolute amplitudes and corresponding TFx for cases 6 (**A**), 13 (**B**), 40 (**C**), and 12 (**D**). Both SCNA gains and losses are represented as absolute values, determined by |1 – ratio to the median|, for visualization purposes. Clinical progression (*P*) or regression (*R*) at each sampling timepoint is shown.

**Figure 4.**
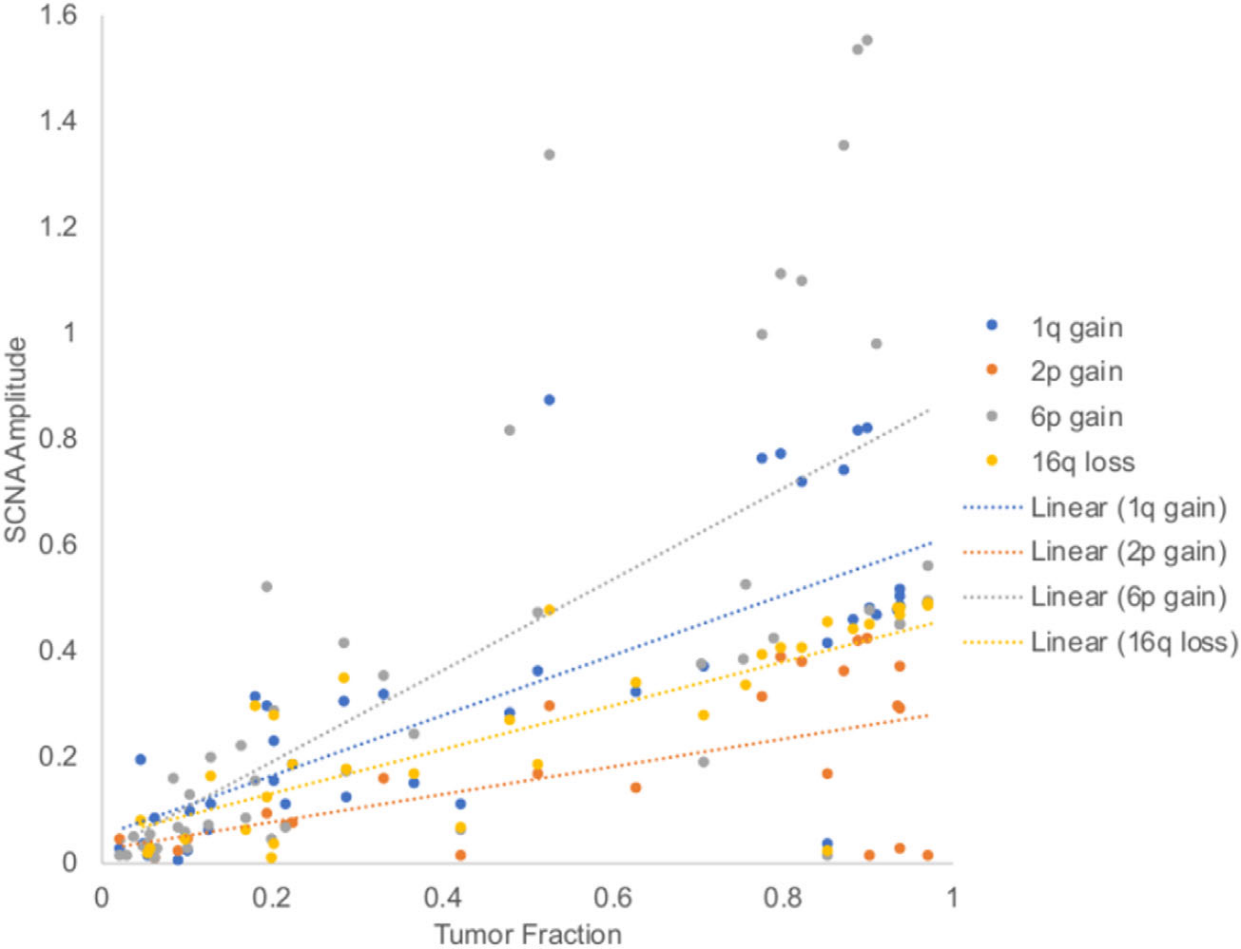
TFx and SCNA absolute amplitudes from aqueous humor cell-free DNA are significantly positively correlated via Pearson correlation. Specifically, TFx was correlated with amplitudes of 6p gain (r(46) = 0.685, *P* < 0.00001), 1q gain (r(40) = 0.763, *P* < 0.00001), 16q loss (r(33) = 0.800, *P* < 0.00001), and 2p gain (r(23) = 0.588, *P* = 0.002). Values are shown as absolute amplitude irrespective of a gain or loss.

The genomic profiles generated by our established protocol (from which SCNA amplitudes were determined^8^^,^^9^) were concordant with the genomic profiles generated by ichorCNA software (Fig. 2), the standard tool used to estimate cfDNA TFx in blood-based liquid biopsies.^23^ Overall, changes in TFx were associated with similar changes in RB SCNA amplitudes (Figs. 3A–C). Specifically, TFx was significantly positively correlated with the amplitudes of 6p gain (r(46) = 0.685, *P* < 0.00001), 1q gain (r(40) = 0.763, *P* < 0.00001), 16q loss (r(33) = 0.800, *P* < 0.00001), and 2p gain (r(23) = 0.588, *P* = 0.002) (Fig. 4).

An exception to this trend can be seen in Case 12 (Fig. 3D). Case 12 was a 13-month-old female who presented with bilateral RB, indicative of a germline mutation in the *RB1* gene. The right eye presented as group C/stage cT2b RB and required three IVM injections for tumor seeding. Standard blood testing for germline *RB1* mutations revealed a deletion in chromosome 13q (13q14.2-q31.1). At the time of the third injection, a large increase in TFx was seen despite stable or decreased SCNA amplitudes (Fig. 3C). This increase in TFx coincided with massive retinal necrosis secondary to IVM toxicity.

## Discussion

Recent years have demonstrated an explosion of scientific reports on the use of cfDNA in serum-based liquid biopsies for nonocular cancers.^12^^–^^16^ However, only recently have innovations in RB therapy^19^ and AH sampling made the detection and analysis of in vivo RB cfDNA possible.^8^^–^^10^^,^^19^ In 2018 we used our established protocol of AH-based SCNA analysis to demonstrate that the amplitudes of SCNAs in two separate RB cases correlated with clinical tumor response.^8^ Now with additional cases, we continue to show an association between SCNA amplitude and clinical progression or regression of disease. There are multiple potential explanations for the relationship between SCNA amplitude and clinical intraocular findings. SCNAs are particularly common genetic occurrences in RB tumor cells (and cancer in general),^22^ so as a tumor responds to chemotherapy and the cancerous cells that contain SCNAs are eliminated, an apparent normalization of chromosomal alterations would be expected.^8^ However, the amplitude of SCNAs is not an independent property; the magnitude of an alteration is confounded by the overall amount of tumor DNA in a sample,^8^ as reflected by the significant correlation between TFx and SCNA amplitudes in our samples. Thus an apparent decrease in SCNA amplitude could suggest not only a decrease in the actual copy number inherent to the tumor but also an overall reduction in tumor-derived DNA (TFx). Furthermore, measuring individual SCNAs does not fully capture the overall genomic landscape of a tumor and therefore cannot account for the heterogeneity or subclonality that may be present within a sample.

Unlike individual SCNA measurements, TFx is a more comprehensive measure of tumor burden by representing the amount of tumor-derived cfDNA relative to the total cfDNA in a sample. Our current platform, based on analyses by Baslan et al.,^20^^,^^21^ can identify SCNAs present within AH samples; however, absolute TFx values are not inferred from our platform alone. In recent years, ichorCNA has become a standard tool for measuring cfDNA TFx in the blood.^12^^,^^13^^,^^23^ The software considers the entire SCNA landscape of cfDNA—while accounting for subclonality—to estimate the fraction of tumor-derived genetic material.^23^ Using ichorCNA, studies of prostate^12^ and lung^13^ cancer have both demonstrated associations between blood-based TFx and tumor dynamics whereby increases in TFx correspond to increased tumor burden and decreases in TFx correspond to regression of disease. When ichorCNA was applied to AH, we found a similar association between TFx and clinical therapeutic response for RB. Additionally, ichorCNA genomic profiles and corresponding TFx estimates correlated with the genomic profiles and SCNA amplitude trends determined by our established AH platform. Although this correlation is expected—because TFx estimates are determined on the basis of the presence of SCNAs in a sample—it has never before been demonstrated in the setting of an AH-based liquid biopsy. Therefore our findings suggest that ichorCNA may have viable clinical utility not only in blood-based liquid biopsies but for the aqueous humor as well.

There can be discordance between TFx changes and clinical response to therapy. Choudhury et al.^12^ theorize that short-term stresses to tumor cells (for example, the administration of chemotherapy) can lead to bursts of cfDNA release—even in the setting of overall clinical regression—resulting in unexpectedly high TFx values. In our Case 12, the observed spike in TFx occurred after the initiation of IVM; therefore it is possible that damage to tumor cells inflicted by chemotherapy led to a temporary fluctuation in cfDNA within the AH resulting in an increased TFx. Additionally this spike coincided with massive retinal necrosis—a rare but known side effect of IVM.^26^ Case 12 had a largescale germline deletion of 13q on standard *RB1* testing, so both the tumor *and* necrotic retinal cells likely contained a 13q loss. Because ichorCNA detects copy number alterations irrespective of whether they are of somatic or germline origin, it is possible that a germline 13q loss in cfDNA from the necrotic retina contributed to a falsely elevated TFx estimate despite overall clinical regression.

While our findings suggest that a strong relationship exists between TFx and RB clinical activity, it should be emphasized that *relative* changes in TFx seem to be more informative of therapeutic response than absolute values. Every sample from case 28, for example, had much lower TFx absolute values than samples from case 35, as shown in Table 1. However, case 35 demonstrated a *decrease* in TFx that corresponded with clinical regression, whereas case 28 demonstrated an *increase* in TFx that corresponded with clinical progression (even though the absolute TFx values remained lower than in case 35 at all times). Although no single, absolute TFx “cut-off” could be associated with clinical progression versus regression, we demonstrated that a TFx increase (relative to baseline) of 15% or more was *highly* predictive of disease progression—and that these TFx increases relative to baseline were even more predictive of disease progression than increases between immediately sequential samples. Even for Case 15—in which an interval of more than one year passed between its first and last samples—clinical progression at the time of final sampling corresponded to a nearly 8.5-fold increase in TFx relative to the initial sample, suggesting that large TFx increases could signal underlying disease progression irrespective of the time between samples. Larger prospective studies of longitudinal AH sampling are necessary to clarify the role of TFx as an indicator of intraocular disease activity in the clinical setting.

The recent development of ichorCNA has facilitated efficient and cost-effective estimations of TFx from low-coverage sequencing data. However, because ichorCNA calculates TFx based on SCNAs detected within a sample, the ability of ichorCNA to accurately determine TFx in samples *without* SCNAs (i.e., with flat genomic profiles) is inherently limited.^23^ Most retinoblastoma tumors contain at least one chromosomal alteration, as we and others have shown.^8^^,^^22^ Nevertheless, not all RB tumors have SCNAs.^8^ In our study, nearly one third of RB eyes were excluded from final analysis because of a lack of SCNAs and corresponding low TFx estimates throughout sampling. A very low TFx calculation from ichorCNA could indicate either (1) that there is no detectable tumor-derived cfDNA in the sample or (2) that tumor cfDNA *is* present but cannot be measured by ichorCNA due to a lack of SCNAs.^23^ Thus a low TFx value from ichorCNA with a corresponding lack of SCNAs *cannot* necessarily exclude the possibility that tumor DNA is still present in the eye. In the future, optimizing our methods of TFx estimation for eyes without SCNAs would be useful to broaden the application of longitudinal TFx monitoring to all RB patients. Because all RB tumors (with few exceptions^27^) contain somatic mutations in the *RB1* gene, calculations based on the detection of *RB1* pathogenic variants could be an alternative approach for estimating TFx in the absence of SCNAs. Although we^9^ and an independent group^28^ have demonstrated that the *RB1* gene is detectable within the AH, studies are ongoing to develop an *RB1*-based TFx pipeline.

Repeated AH sampling is not yet a part of standard RB management; however, our findings imply potential value in analyzing the AH before, during, and after treatment. Some possible clinical applications of longitudinal AH monitoring include: 1) detection of TFx decline as a marker of therapeutic response, 2) identification of tumors that are resistant to therapy (i.e., if a TFx decline is not seen), and 3) early detection of disease recurrence, before obvious clinical progression.^12^^,^^13^ Currently, our AH liquid biopsy protocol is confined to the research setting with AH sampled during IVM injections, or immediately after enucleation. However, since we have demonstrated that changes in AH cfDNA properties coincide with clinical response, there may be clinical value in revising the current protocol to include routine sampling of AH 1) at diagnosis, 2) after the completion of systemic or intraarterial chemotherapy to evaluate for adequate response, and 3) after completion of consolidative therapy to monitor for residual or recurrent malignant disease. Our findings indicate that the measurement of longitudinal AH cfDNA dynamics may provide a novel way to objectively monitor tumor activity throughout the management of RB. However, prospective studies examining AH at diagnosis and after AH sampling throughout treatment are required before we can directly apply this to clinical interventions in patients.

In summary, our study demonstrates that changes in both SCNA amplitude and TFx correspond to clinical progression and regression of disease over time, with TFx representing a comprehensive measure of tumor DNA within a sample. Although ichorCNA is limited with regard to RB tumors that lack SCNAs, technologies continue to improve and allow more cost-effective and efficient determinations of tumor fraction.^23^ With a better understanding of in vivo RB genetics and tumor dynamics, there is potential for improved clinical decision making and more personalized, precision-based patient care. If validated in future studies, TFx in the AH could potentially serve as a clinically relevant biomarker of RB therapeutic response.

## Supplementary Material

Supplement 1

Supplement 2
